# Coronary artery aneurysm and thrombosis associated myocardial infarction in a 24-year-old with multisystem inflammatory syndrome and human immunodeficiency virus: a case report

**DOI:** 10.1093/ehjcr/ytag242

**Published:** 2026-04-06

**Authors:** Abbey Grbac, Olivia Slifirski, Vincenzo Somma, David Bridge, Matthew Brooks

**Affiliations:** Department of Cardiology, The Royal Melbourne Hospital, 300 Grattan St, Parkville, VIC 3050, Australia; Department of Cardiology, The Royal Melbourne Hospital, 300 Grattan St, Parkville, VIC 3050, Australia; Department of Cardiology, The Royal Melbourne Hospital, 300 Grattan St, Parkville, VIC 3050, Australia; Department of Cardiology, The Royal Melbourne Hospital, 300 Grattan St, Parkville, VIC 3050, Australia; Department of Cardiology, The Royal Melbourne Hospital, 300 Grattan St, Parkville, VIC 3050, Australia

**Keywords:** Myocardial infarction, Coronary artery aneurysm, Multisystem Inflammatory Syndrome in Children, Human Immunodeficiency Virus, Case report

## Abstract

**Background:**

Multisystem Inflammatory Syndrome in Children (MIS-C) and Human Immunodeficiency Virus (HIV) are both systemic inflammatory diseases that rarely result in coronary artery aneurysms (CAA). Interventional treatment and management of CAA pose a challenge to clinicians with no current guideline recommendations. We present the first case of CAA with thrombus and myocardial infarction in the setting of both MIS-C and HIV.

**Case summary:**

24-year-old male with a background of MIS-C and HIV presented with chest pain, anterior ST-segment elevation and troponin I > 50 000 ng/L, complicated by three cardiac arrests with pulseless ventricular tachycardia requiring direct cardioversion. Angiogram showed left anterior descending (LAD) CAA measuring 8 × 9 mm with an occlusive thrombus in the proximal LAD. Initial angioplasty and thrombectomy attempts were unsuccessful. Intracoronary thrombolysis was administered, followed by further balloon inflations and thrombus aspiration with restoration of TIMI3 flow.

**Discussion:**

This case is significant as it highlights a novel presentation of myocardial infarction in a young adult. We propose a high index of suspicion of thrombotic events in all patients with known MIS-C or HIV to ensure timely identification and management. We also highlight the complexity in decision-making in interventional and long-term management of CAA with large intracoronary thrombosis and the need for further research into optimal approaches.

Learning points

- Giant coronary aneurysms and subsequent intracoronary thrombosis are rare, with most occurring in the setting of medium vessel vasculitis
- This is the first case of thrombotic coronary artery aneurysm in the setting of Multisystem Inflammatory Syndrome in Children (MIS-C) and Human Immunodeficiency Virus (HIV)
- Clinicians should have a high suspicion of thrombotic events in patients with MIS-C or HIV to ensure early identification and treatment

## Introduction

Coronary artery aneurysms (CAAs) are commonly caused by atherosclerosis in adults and Kawasaki disease in paediatric patients. Two novel aetiologies are Multisystem Inflammatory Syndrome in Children (MIS-C) and Human Immunodeficiency Virus (HIV).^1,2^ We present the first case of CAA with thrombus and myocardial infarction (MI) without atherosclerosis in the setting of MIS-C and HIV. This case not only highlights a rare cause of CAAs but also the challenges in management, with further research required.

## Summary figure

**Figure:**
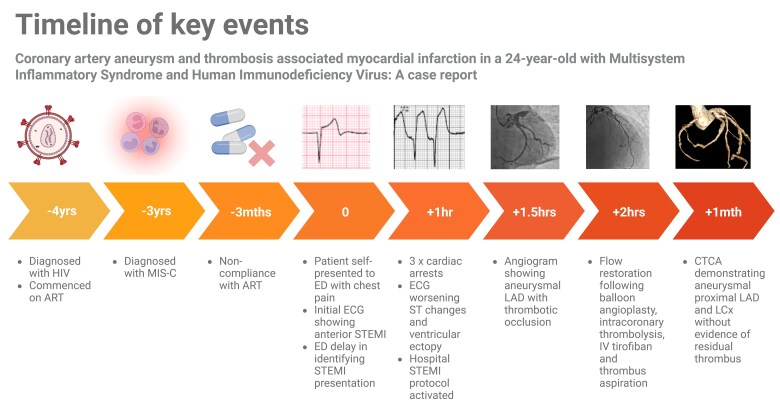


## Case report

A 24-year-old man self-presented to the emergency department with chest pain. His history was significant for MIS-C diagnosed in April 2022 following SARS-CoV-2 infection and HIV diagnosed in September 2021, initially on antiretroviral therapy (ART); however, non-compliant for 3-months prior to this presentation.

**Figure 1:**
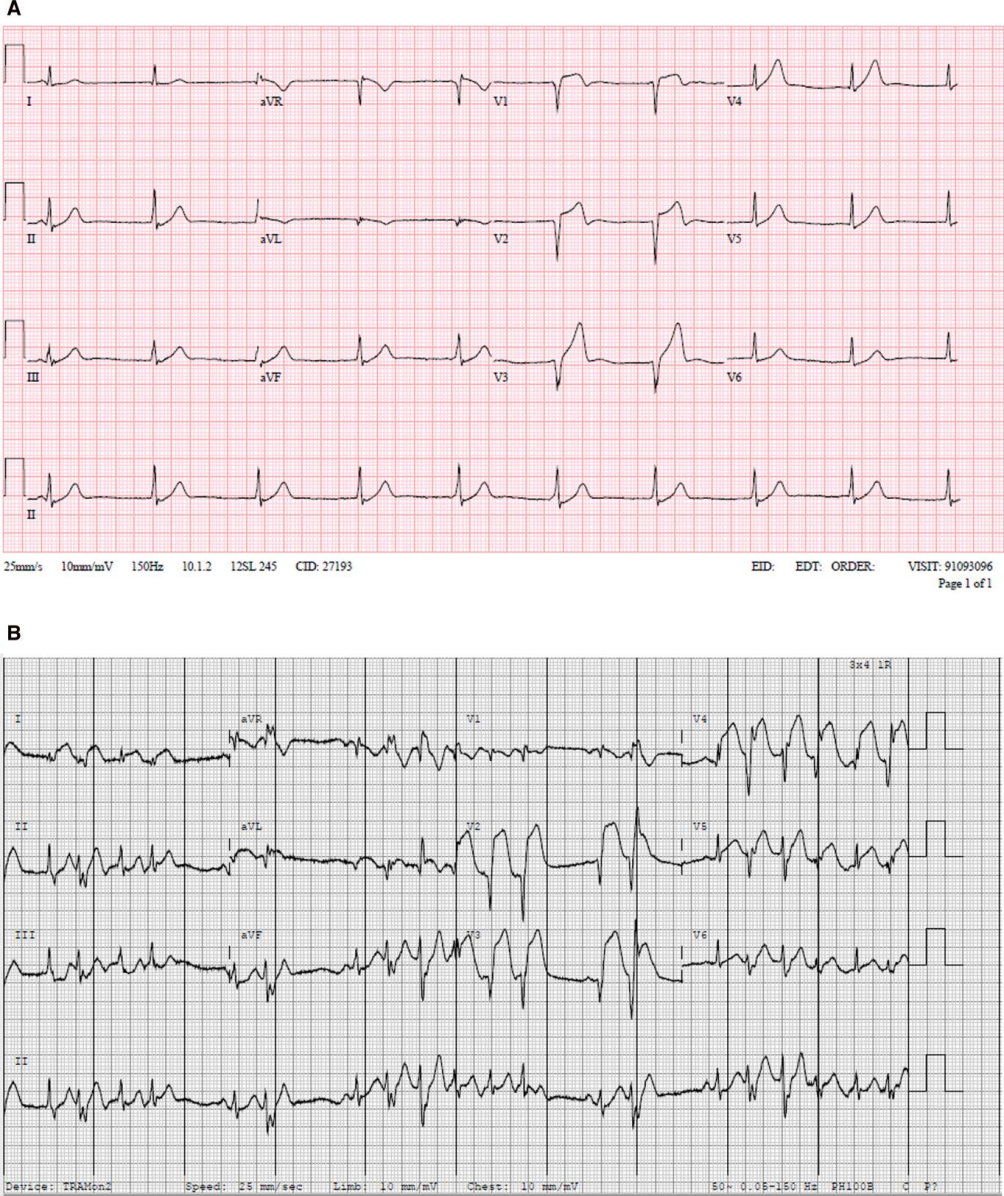
(*A*) Admission 12-lead electrocardiogram (ECG) demonstrating anteroseptal ST-segment elevation and hyperacute T waves. (*B*) ECG following successful resuscitation demonstrates worsening of the ST-segment elevation with frequent ventricular ectopy.

On initial examination, respiratory rate was 18bpm, saturations 100% on air, heart rate 61bpm, blood pressure 101/83 mmHg, temperature 36.1°C, he had dual heart sounds without murmurs, with no signs of heart failure, rashes, lymphadenopathy, or oropharyngeal oedema. Despite the initial electrocardiogram (ECG) demonstrating ST-segment elevation in leads V1–3 fulfilling criteria for anteroseptal ST elevation myocardial infarction (STEMI) (*Figure 1A*), identification and activation of the catheterisation laboratory weas delayed. He developed paroxysmal ventricular arrhythmia, resulting in loss of output on three occasions, requiring cardioversion. Following resuscitation, the ECG demonstrated worsening ST segments with frequent ventricular ectopy (*Figure 1B*). Aspirin, amiodarone and intravenous heparin were administered. The patient was transferred to the catheterisation laboratory conscious and requiring no organ support.

**Figure 2:**
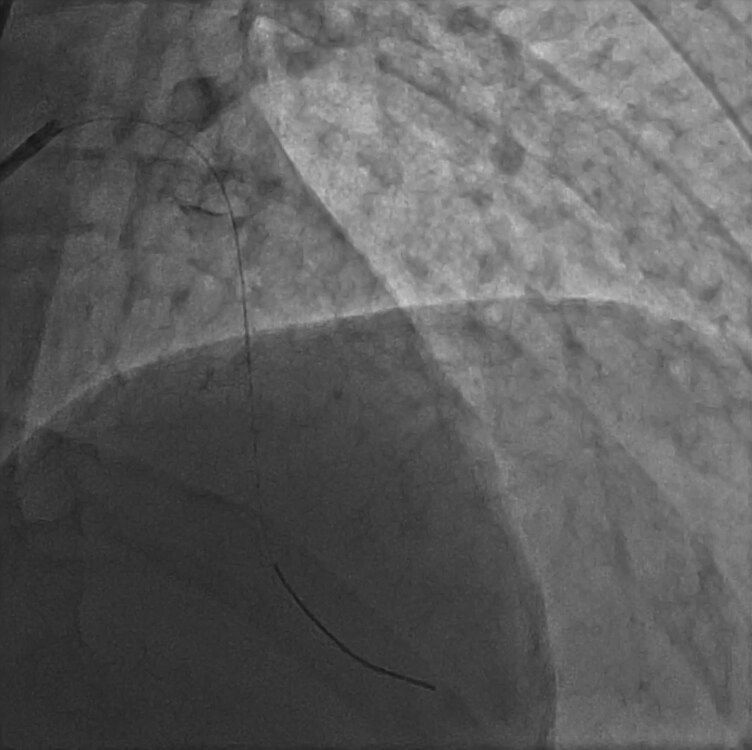
Coronary angiogram cine.

**Figure 3:**
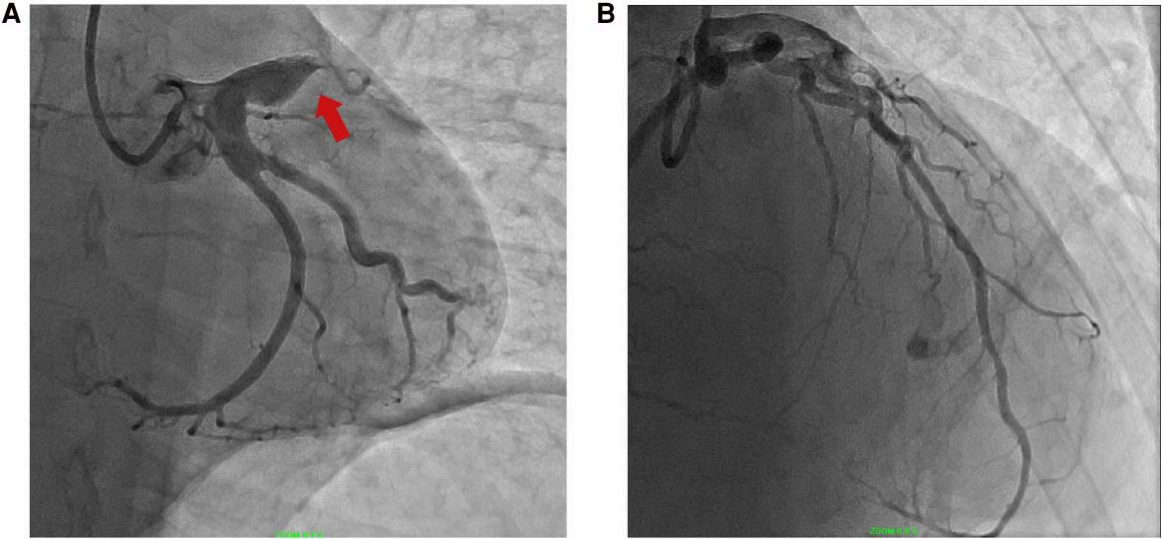
(*A*) left anterior oblique (LAO) caudal projection of the left coronary system with left anterior descending (LAD) significantly aneurysmal proximally (arrow) and occluded, and left circumflex proximally aneurysmal with flow. (*B*) Posteroanterior (PA) cranial projection of the left coronary system showing flow restoration to the LAD.

**Figure 4:**
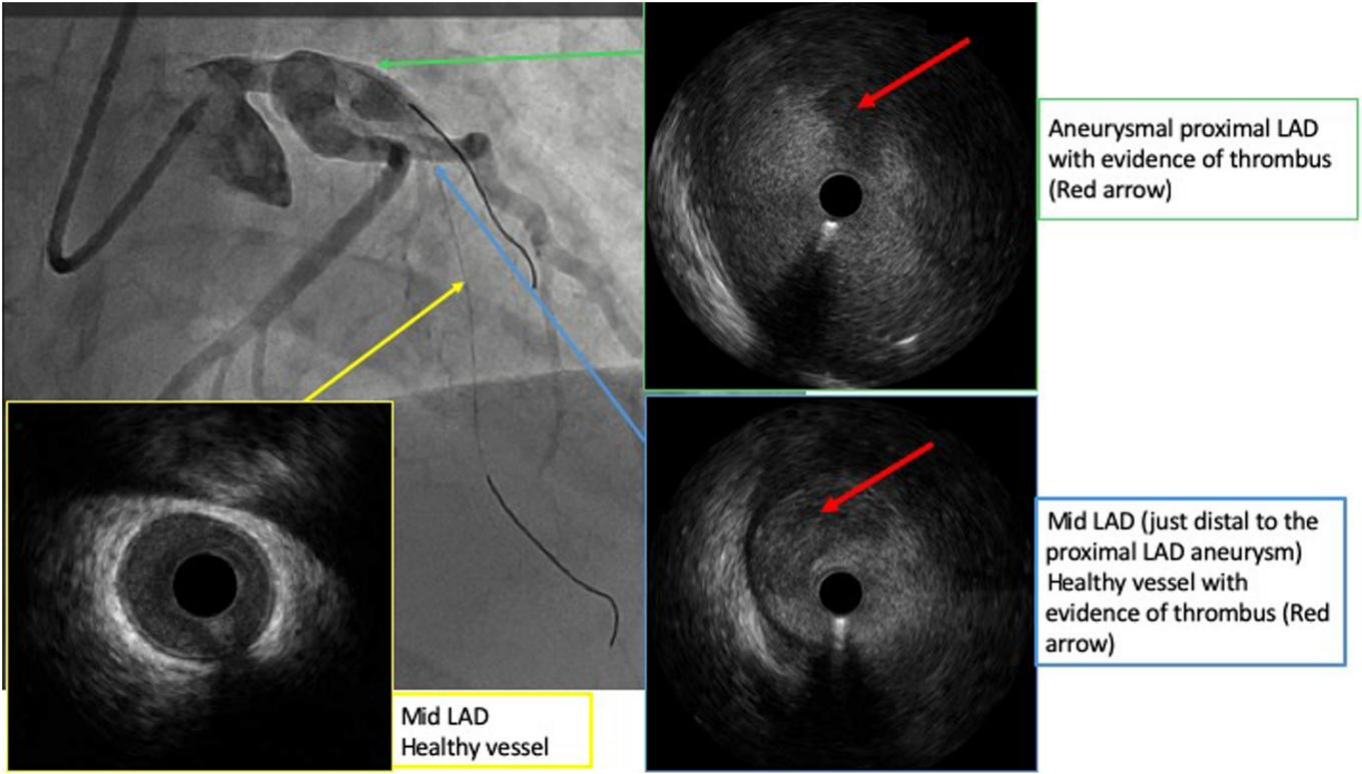
Intravascular ultrasound (IVUS) stills demonstrating a true aneurysm with thrombus without atherosclerosis.

Coronary angiogram (cine available in *Figure 2*) demonstrated a severely aneurysmal proximal left anterior descending (LAD) artery with acute occlusion secondary to thrombus (*Figure 3A*). The left circumflex artery (LCx) was dominant, unobstructed and aneurysmal proximally. The right coronary artery was non-dominant and free of disease. The LAD was successfully wired with a workhorse coronary wire. Multiple balloon inflations, intracoronary nitrates, intravenous tirofiban and bail-out thrombus aspiration (via thrombectomy catheter and GuideLiner) were attempted; there remained no flow in the LAD. Intravascular ultrasound (IVUS) (images available *Figure 4*) was performed with LAD lumen approximated to be at 8–9 mm without atherosclerosis present and confirmed a high volume of thrombus within the aneurysmal segment (*Figure 3B*). Given balloon angioplasty, GPIIb/IIIa inhibitor and thrombectomy were unsuccessful, systemic thrombolysis was considered. However, given local experience (including a dedicated intracoronary thrombolysis protocol) and ability to deliver thrombolysis directly to the area, we opted for this method over systemic therapy with alteplase (two doses of 2.5milligrams) administered. Following this, further balloon inflations and thrombus aspiration to the mid-LAD resulted in the removal of a large thrombus with restoration of flow with Thrombolysis in Myocardial Infarction (TIMI) grade 3 (*Figure 3C*). Stenting was not performed due to the significant size discrepancy, causing a diameter mismatch in the proximal LAD with high risk of malapposition as well as residual thrombus and no clear culprit atherosclerotic lesion once flow was restored. Coronary artery bypass grafting was not required, if coronary intervention failed, the patient was located in a hospital with Cardiothoracic surgery backup. The procedure was well tolerated with no immediate complications, and the patient was transferred to the cardiac coronary unit.

**Figure 5:**
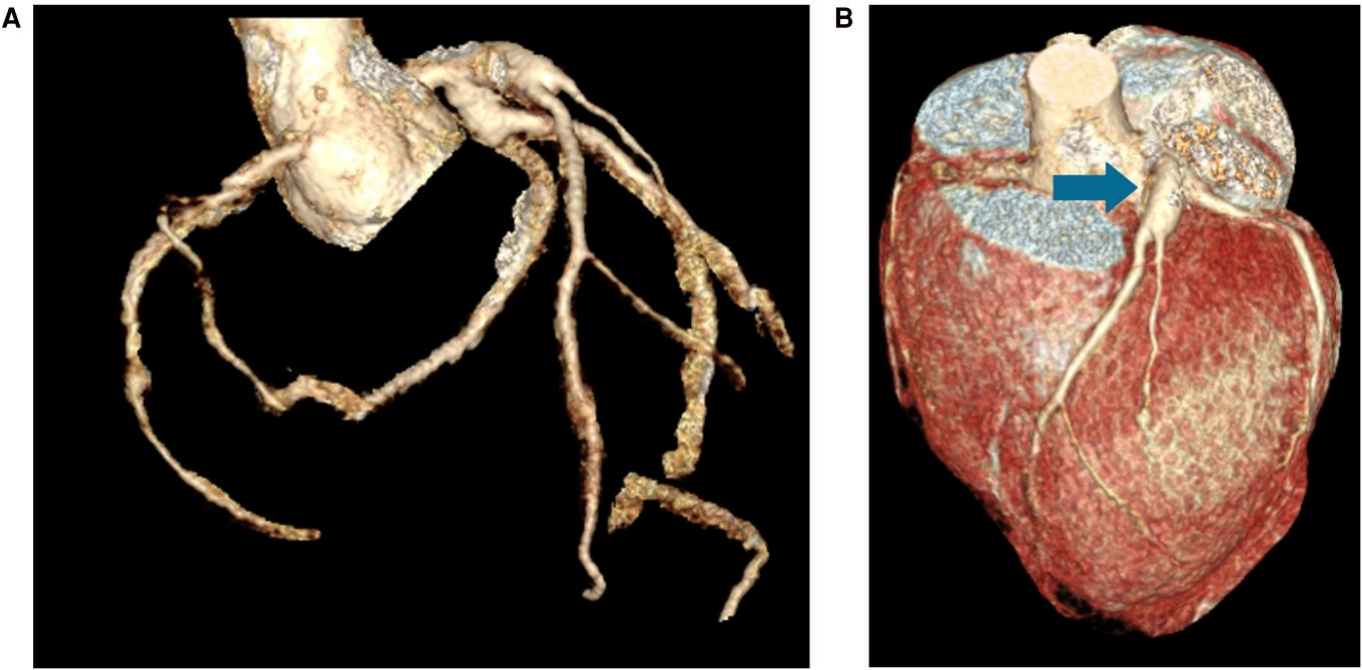
(*A*) 3D reconstruction of coronary arteries with aneurysmal proximal left anterior descending (LAD) (8 × 9 mm luminal diameter) and proximal circumflex vessel (7 × 7 mm diameter). (*B*) 3D reconstruction of the heart with aneurysmal proximal LAD marked by an arrow.

On day 0, his troponin I peaked at >50 000 ng/L, CRP 24.7 mg/L, ESR 3 mm/hr, INR 1.0, APTT 114 (on heparin), fibrinogen 2.5, PT 14.2, ferritin 227. HIV viral load was 190 561 copies/mL, and absolute CD4 count was 0.28 × 10^9^/L. Alternative diagnoses such as medium vessel vasculitis, connective tissue disease, post-infectious or drug-induced vasculitis were sought, with relevant panels performed in consultation with Infectious Diseases and Immunology experts. The results of all tests, including laboratory and angiographic, coupled with patient history, examination findings and specialist team input, found no other cause for the patient's presentation other than the background MIS-C and HIV. Subsequent transthoracic echocardiogram (TTE) indicated a left ventricular ejection fraction (LVEF) of 32% secondary to moderate segmental systolic dysfunction. The patient was established on anticoagulation with intravenous heparin and discharged on dual antiplatelets, apixaban and commenced on ART. The patient was treated with ‘triple therapy’ for 2 weeks, followed by apixaban and a single antiplatelet with a planned 12-month treatment course, followed by apixaban lifelong. Computer Tomography Coronary Angiogram (CTCA) (*Figure 5*) at 4 weeks showed aneurysmal proximal LAD and LCx without evidence of residual thrombus. He was monitored in the community with serial TTEs with plans for further aneurysmal surveillance with CTCA. LVEF improved to 42% in setting guideline-directed medical therapy with NYHA Class I symptoms on the last review. He has had no further arrhythmic or ischaemic events so has not undergone further testing. He continues to be regularly followed up by Infectious Diseases, with compliance to ART and an undetectable HIV viral load.

## Discussion

The incidence of giant coronary aneurysms (GCAs), with a diameter exceeding 8 mm and subsequent intracoronary thrombosis, is 0.02%.^3^ MIS-C is a hyperinflammatory condition similar to Kawasaki disease, with 8–24% of patients developing CAA.^1,4^ Coronary aneurysms occurring in MIS-C are usually small, with 80% regressing within 30-days of treatment with immunoglobulins and steroids. The exception, however, is GCAs, which increase in size.^4^ There are case reports of delayed GCAs detected after the index presentation of MIS-C and complications such as MI occurring months to years after the initial diagnosis.^5–9^

Although aneurysms are a complication of HIV, the incidence of CAA is lower, with an occurrence of 0.8–4.9%.^10,11^ A common cardiac manifestation of HIV is early coronary artery disease (CAD), with prevention via aggressive risk factor modification and initiation of ART. Our patient lacked risk factors for CAD, and an atherosclerotic lesion was not detected on angiogram with IVUS. The pathophysiology of aneurysm formation is chronic inflammation resulting in coagulation abnormalities, endothelial dysfunction and ectasia with an increased risk of MI.^3^ There are case reports of HIV patients both on and in the absence of ART developing STEMI in the setting of CAAs and/or thrombus.^12,13^ Our case is unique as it is the first reported case of CAA associated with both MIS-C and HIV, resulting in thrombotic coronary occlusion. We propose a high index of suspicion of thrombotic MI in patients with a background of MIS-C or HIV.

This case highlights the complexity in clinician decision-making, including strategies for high thrombus burden and significant vessel diameter mismatch during the index procedure. As well as long-term management issues like anticoagulation and follow-up. We based our management decisions on guidelines for patients with similar conditions, such as Kawasaki disease, in addition to consensus guidelines based on the available literature for MIS-C and HIV, as well as trials in patients with CAAs with MI. Long-term anticoagulation with apixaban was an example of this combined decision-making process.^1,14^ When available, we followed standardized guideline recommendations such as secondary prevention following MI. More research is required to investigate optimal interventional techniques and management strategies.

## Limitations

This report is based on a single patient's presentation, so it lacks generalisability and the ability to prove cause and effect. Although the patient had a background of MIS-C and HIV, we can not definitively prove the patient's presentation was due to these conditions, with a delay from the diagnosis of these conditions to the acute presentation.

## Conclusion

This case represents a unique cause of MI. Early identification of MI in patients with MIS-C or HIV is important as these patients often lack traditional characteristics of CAD. Given the complexities in management, high-quality research is required to guide optimal medical, interventional and surveillance approaches.

## Supplementary Material

ytag242_Supplementary_Data

## Data Availability

Data supporting the findings of this study are available within the main manuscript.

## Lead author biography

Dr Abbey Grbac studied a Bachelor of Science at the University of Melbourne, majoring in Anatomy, before completing a Master's in Medical Radiation at Monash University in 2017. She completed her Medical Doctorate at the University of Melbourne in 2021, awarded with Distinction. Abbey is in her final year of Basic Physician’s Training at the Royal Melbourne Hospital.

## Notes

**Consent:** The patient gave informed consent for the material presented to appear in the publication.
